# Thyroid Hormone-Mediated Growth and Differentiation of Growth Plate Chondrocytes Involves IGF-1 Modulation of β-Catenin Signaling

**DOI:** 10.1002/jbmr.5

**Published:** 2009-12-21

**Authors:** Lai Wang, Yvonne Y Shao, R Tracy Ballock

**Affiliations:** Orthopaedic Research Center, Departments of Orthopaedic Surgery and Biomedical Engineering, The Lerner Research Institute, The Cleveland Clinic Foundation Cleveland, OH, USA

**Keywords:** thyroid hormone, insulin-like growth factor 1, β-catenin, growth plate chondrocytes

## Abstract

Thyroid hormone regulates terminal differentiation of growth plate chondrocytes in part through modulation of the Wnt/β-catenin signaling pathway. Insulin-like growth factor 1 (IGF-1) has been described as a stabilizer of β-catenin, and thyroid hormone is a known stimulator of IGF-1 receptor expression. The purpose of this study was to test the hypothesis that IGF-1 signaling is involved in the interaction between the thyroid hormone and the Wnt/β-catenin signaling pathways in regulating growth plate chondrocyte proliferation and differentiation. The results show that IGF-1 and the IGF- receptor (IGF1R) stimulate Wnt-4 expression and β-catenin activation in growth plate chondrocytes. The positive effects of IGF-1/IGF1R on chondrocyte proliferation and terminal differentiation are partially inhibited by the Wnt antagonists sFRP3 and Dkk1. T_3_ activates IGF-1/IGF1R signaling and IGF-1-dependent PI3K/Akt/GSK-3β signaling in growth plate chondrocytes undergoing proliferation and differentiation to prehypertrophy. T_3_-mediated Wnt-4 expression, β-catenin activation, cell proliferation, and terminal differentiation of growth plate chondrocytes are partially prevented by the IGF1R inhibitor picropodophyllin as well as by the PI3K/Akt signaling inhibitors LY294002 and Akti1/2. These data indicate that the interactions between thyroid hormone and β-catenin signaling in regulating growth plate chondrocyte proliferation and terminal differentiation are modulated by IGF-1/IGF1R signaling through both the Wnt and PI3K/Akt signaling pathways. While chondrocyte proliferation may be triggered by the IGF-1/IGF1R-mediated PI3K/Akt/GSK3β pathway, cell hypertrophy is likely due to activation of Wnt/β-catenin signaling, which is at least in part initiated by IGF-1 signaling or the IGF-1-activated PI3K/Akt signaling pathway. © 2010 American Society for Bone and Mineral Research.

## Introduction

During longitudinal bone growth, growth plate chondrocyte proliferation and differentiation are regulated by a variety of endocrine and paracrine hormones and growth factors. Local paracrine regulators that are essential for normal bone formation include bone morphogenetic proteins (BMPs), Wnts, parathyroid hormone-related protein (PTHrP), Indian hedgehog (Ihh), and insulin-like growth factor 1 (IGF-1).(1) PTHrP and Ihh interact in a negative-feedback loop to regulate the transition from proliferation to differentiation in growth plate chondrocytes. Wnt and BMP signaling are cooperative signaling pathways that promote chondrocyte terminal differentiation. Several investigators, including our laboratory, have identified Wnt-4 as a positive regulator of terminal differentiation of growth plate chondrocytes.(2–4)

IGF-1 has been shown to be an important regulator of endochondral ossification.(5–11) IGF-1 stimulates proliferation of resting-zone chondrocytes, augments chondrocyte hypertrophy, and promotes longitudinal bone growth.(5–7) The effects elicited by the systemic administration of IGF-1 to hypophysectomized rats suggest that IGF-1 stimulates growth plate chondrocytes at all stages of differentiation.(5) Local infusion of IGF-1 in the rabbit tibial growth plate results in an acceleration of the tibial growth rate and an increased number of proliferative and hypertrophic growth plate chondrocytes.(6) In fetal mouse metatarsals, IGF-1 induces cell proliferation and hypertrophy in the growth plate.(7) IGF-1 signaling is also required to maintain the Ihh-PTHrP loop during skeletogenesis. In fetal *Igf1*^*−/−*^ mice, expression of Ihh was reduced in long bones, whereas expression of PTHrP was increased.(8) *Igf1* null mice exhibit severe prenatal growth plate defects and a subnormal postnatal growth rate.(9,10) The tibial growth plate in the *Igf1* null mice exhibits an expanded resting zone and a significantly reduced hypertrophic zone.(11)

IGF-1 signals via the type 1 IGF receptor (IGF1R), which is expressed in the proliferating and prehypertrophic zone chondrocytes of growth plate,(12) which is similar in localization to PTHrP and PTH/PTHrP receptor expression.(8) Compared with *Igf1*^*−/−*^ mice, null mutants for the *Igf1r* gene exhibit even more severe growth retardation.(9) The growth plates of *Igf1r*^*−/−*^ mouse embryos show delayed chondrocyte maturation and poor formation of primary ossification centers.(10) The action of IGF-1 within the growth plate is also regulated by IGF-binding proteins (IGFBPs), which bind IGF with high affinity and potentially can either inhibit or enhance IGF activity depending on the complement of IGFBPs present.(13)

Thyroid hormone is a systemic factor that potently regulates skeletal maturation in the growth plate. Thyroid hormone receptor α (TR-α) is essential for regulating the process of endochondrial ossification. *TRα1*^*PV/+*^ mice, which are lacking *TRα1*, exhibit skeletal hypothyroidism with delayed endochondral ossification and severe postnatal growth retardation.(14) *TRα1*^*PV/+*^ mice also have impaired *Igf1r* expression and IGF-1 signaling in the growth plate, suggesting that the IGF1R is a physiologic target for thyroid hormone action in the growth plate.(14)

β-Catenin signaling also has been recognized as an important signal-transduction pathway in regulating terminal differentiation of growth plate chondrocytes. Inhibition of β-catenin signaling in *Col2a1-ICAT* transgenic mice results in reduced chondrocyte proliferation and differentiation, delayed formation of the secondary ossification center, and reduced skeletal growth.(15) Our previous studies have shown that thyroid hormone interacts with the Wnt/β-catenin signaling pathway in regulating the terminal differentiation of growth plate chondrocytes.(4)

GSK-3β is a negative regulator of the canonical Wnt/β-catenin pathway.(16) β-Catenin is phosphorylated by active GSK-3β and targeted for degradation. Wnt ligands inhibit the formation of the axin/APC/GSK3 complex and block β-catenin phosphorylation by GSK-3β, resulting in the stabilization of β-catenin.

GSK-3β is also involved in the IGF-1 signaling pathway. Phosphatidylinositol-3-kinase (PI3K) is an important signal transducer of responses to IGF-1 signaling. Akt is a downstream target of PI3K, and can inactivate GSK-3β by phosphorylation on serine 9.(17) *Igf1*^*−/−*^ mice exhibit hypophosphorylated GSK-3β in the tibial growth plates.(11) Raucci and colleagues reported that IGF-1 signals induce Akt phosphorylation and promote osteoblast differentiation, and cells expressing active Akt have increased levels of stabilized β-catenin.(18) IGF-1 also regulates the location, stability, and transcriptional activity of β-catenin in cancer cells.(19)

These observations support the concept of crosstalk between IGF-1 and Wnt signaling pathways in regulating growth plate chondrocyte differentiation. IGF-1 signals may potentiate the biologic functions of Wnt signaling by modulating β-catenin signaling through PI3K/Akt pathway. The purpose of this study was to test the hypothesis that thyroid hormone regulates proliferation and differentiation of growth plate chondrocytes through IGF-1 modulation of the activity of β-catenin signaling in growth plate chondrocytes.

## Materials and Methods

### Cell culture

Chondrocytes were isolated from the resting zone of the distal femoral growth plate of 2-day-old neonatal Sprague-Dawley rats by sequential digestion in trypsin/EDTA (Invitrogen, Carlsbad, CA, USA) for 1 hour at 37°C, followed by 0.3% collagenase type I (Worthington, Lakewood, NJ, USA) for 4 hours at 37°C.(20) Cells were resuspended in DMEM/F12 medium (Invitrogen) supplemented with a defined medium supplement (ITS+1, Sigma, St. Louis, MO, USA) and plated in monolayer or in 3D pellet cultures.(4) Triiodothyronine (T_3_, Sigma) was added to the medium at a concentration of 100 ng/mL. Recombinant mouse IGF-1, Frzb/sFRP3, and Dkk1 (R&D Systems, Minneapolis, MN, USA) were used at concentrations of 50, 100, and 100 ng/mL, respectively. The IGF1R inhibitor picropodophyllin (PPP), the PI3K inhibitor LY294002, and the Akt inhibitor Akti1/2 were purchased from Calbiochem (La Jolla, CA, USA) and used at concentrations of 1, 20, and 1 µM, respectively.

An adenovirus encoding the IGF1R (Ad-IGF1R) was kindly supplied by Dr Delafontaine (Tulane University School of Medicine, New Orleans, LA, USA). The adenovirus was used at a multiplicity of infection (MOI) of 50. A structurally similar adenovirus containing the Cytomegalovirus (CMV) promoter was used as a negative control. Growth plate chondrocytes were infected with adenoviral vectors in 60-mm dishes. Twenty-four hours after infection, the cells in monolayer were trypsinized and maintained as pellet cultures.

### Quantitative real-time PCR

Total RNA was isolated from cultured growth plate chondrocytes using the RNeasy Kit (Qiagen, Valencia, CA, USA) according to the manufacturer's instructions. Reverse transcription was performed using random primers and Superscript III DNA polymerase (Invitrogen). Real-time polymerase chain reaction (PCR) reactions were conducted in an ABI Prism 7700 Sequence Detection System using SYBR Green PCR core reagents (Applied Biosystems, Foster City, CA, USA). The forward and reverse primers for the amplifications were as follows: IGF-1: 5'-GC-TATGGCTCCAGCATTCG-3' and 5'-AGATCACAGCTCCGGAAGCA-3'; IGF1R: 5'-CCTGGCGTGCTGGTTCTC-3' and 5'-GGCGCGTCCCCCATT-3'; Wnt-4: 5'-AACCGGCGCTGGAACTG-3' and 5'-GGTCCCTTGTGTCACCACCTT-3'; cyclin D1: 5'-CCCACGATTTCATCGAACACT-3' and 5'-GTGCATGTTTGCGGATGATC-3'; Runx2/cbfa1: 5'-TTTAGGGCGCATTCCTCATC-3' and 5'-GGAGGGCCGTGGGTTCT-3'; Col10a1: 5'-GATCATGGAGCTCACGGAAAA-3' and 5'-CCGTTCGATTCCGCATTG-3'; and 18s: 5'-AGTCCCTGCCCTTTGTACACA-3' and 5'-GATCCGAGGGCCTCACTAAAC-3'.

### Cell proliferation assay

Cells from the resting-zone growth plate were plated at a density of 2.5 × 10^3^ cells per well on 96-well plates. Cells were incubated in medium supplemented with or without T_3_, PPP, IGF-1, and Ad-IGF1R for 5 days. Proliferation was assessed by measuring the incorporation of 5-bromo-2'-deoxyuridine (BrdU) into DNA using the BrdU Cell Proliferation Assay Kit (Exalpha Biologicals, Shirley, MA, USA) according to the manufacturer's instructions. The amount of BrdU was quantified spectrophotometrically with a microplate spectrophotometer at a wavelength of 450 nm with reference at a wavelength of 570 nm.

### Immunoblotting

Whole-cell extracts were prepared from cultured chondrocytes using modified RIPA buffer (50 mM Tris-HCl, pH 7.4; 1% NP-40; 0.25% sodium deoxycholate; 150 mM NaCl; 1 mM EDTA; 1 mM PMSF; 1 µg/mL each of aprotinin, leupeptin, and pepstatin; 1 mM Na_3_VO_4_; and 1 mM NaF). An equal amount of protein was separated by 10% SDS-PAGE and transferred onto nitrocellulose membranes. The cellular accumulation of active β-catenin was detected using an antibody against β-catenin (Sigma). Antibody against IGF1R was purchased from Santa Cruz Biotechnology (Santa Cruz, CA, USA). Antibodies against phosphorylated Akt (pAkt) and phosphorylated GSK-3β (pGSK-3β) were obtained from Cell Signaling Technology (Beverly, MA, USA). Anti-β-actin (Sigma) was used as an internal control. The blots were incubated with a horseradish peroxidase (HRP)–conjugated secondary antibody (Santa Cruz). Immunoreactive proteins were visualized by Western blotting chemiluminescence luminol reagent (Santa Cruz).

### Enzyme-linked immunosorbent assay (ELISA)

Secretion of IGF-1 from the pellet cultured growth plate cells into the culture medium was measured 1 to 8 days after addition of 100 ng/mL of T_3_ using a mouse/rat IGF-1 ELISA quantikine kit (R&D Systems, Minneapolis, MN, USA) according to the manufacturer's instructions. The level of IGF-1 in the medium was normalized to the total protein content of each pellet.

### Alkaline phosphatase activity assay

Alkaline phosphatase activity was measured in growth plate chondrocytes in pellet cultures. Pellets were homogenized, and alkaline phosphatase activity was determined as described previously using *p*-nitrophenyl phosphate (Sigma) as a substrate.(20) One unit of alkaline phosphatase was defined as the enzyme activity that liberated 1 µmol of *p*-nitrophenol per 30 minutes at 37°C per milligram of protein.

### Statistical analysis

All data were represented as mean ± SD. Values were assessed by one-way ANOVA with the Bonferroni post hoc test or Student's *t* test at a significance level of *p* < .05.

## Results

### T_3_ upregulates *Igf1r* expression in proliferating and prehypertrophic growth plate cells

**Fig. 1:**
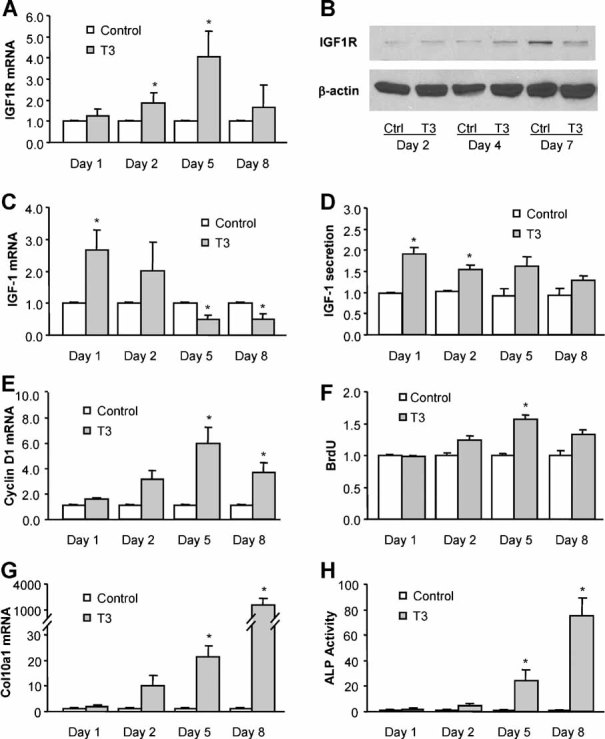
Thyroid hormone treatment increases *Igf1r* expression in rat growth plate chondrocytes. (*A*, *C*) Quantitative real-time RT-PCR analysis of *Igf1r* and *Igf1* mRNA expression in pellet cultures of growth plate chondrocytes treated with 100 ng/mL of T_3_ for 1 to 8 days. The expression in T_3_-treated cells was normalized to the expression in control cells. (*B*) Immunoblotting of IGF1R protein from whole-cell lysates of growth plate chondrocytes treated with or without T_3_ (100 ng/mL) for 2 to 7 days. Actin was used as an internal control. (*D*) Detection of IGF-1 protein in culture medium of growth plate chondrocytes treated with or without T_3_ for 1 to 8 days. (*E*, *F*) *Cyclin D1* mRNA and BrdU incorporation in growth plate cells treated with or without T_3_ for 1 to 8 days. (*G*, *H*) *Col10a1* mRNA and alkaline phosphatase activity in growth plate chondrocytes treated with or without T_3_ for 1 to 8 days. *p < .05 versus control cells.

*Igf1r* mRNA expression was increased in pellet cultures of growth plate cells treated with T_3_ as early as day 2, with the peak increase in expression at day 5 of T_3_ treatment and decreased expression thereafter (Fig. 1*A*). To confirm that the increase in gene expression indeed resulted in a corresponding increase in IGF1R protein, immunoblotting was performed and demonstrated an increase in IGF1R on day 4 of T_3_ treatment, with the level decreasing on day 7 (Fig. 1*B*).

Expression of *Igf1* mRNA in growth plate cells was transiently upregulated by T_3_ treatment on day 1 and started to decrease on day 5 of T_3_ treatment (Fig. 1*C*). IGF-1 protein secreted into the medium from the cultured growth plate cells was increased within the first 2 days of T_3_ treatment (Fig. 1*D*).

Chondrocytes collected from the resting-zone growth plate did not hypertrophy in pellet cultures for at least 2 weeks when treated with basic medium without T_3_ (data not shown). To understand the cell differentiation stage of the growth plate chondrocytes treated with T_3_, the cell proliferation markers *cyclin D1* mRNA and BrdU incorporation, as well as cell hypertrophic markers *Col10a1* mRNA and alkaline phosphatase (ALP) activity, were analyzed in the pellet cultures of growth plate cells treated with or without T_3_ for 1 to 8 days. The results showed that *cyclin D1* mRNA expression and BrdU incorporation peaked on day 5 of T_3_ treatment (Fig. 1*E, F*), and *Col10a1* mRNA expression and ALP activity started increasing on day 5 and reached the highest levels after 8 days of treatment (Fig. 1*G, H*). These data indicate that the cells were predominantly in the proliferating or prehypertrophic stage after 5 days of T_3_ treatment, whereas most cells hypertrophied when treated with T_3_ for 8 days. Upregulation of *Igf1r* expression by T_3_ appeared to occur in growth plate chondrocytes undergoing both proliferation and prehypertrophy.

### IGF1R inhibitor attenuates T_3_-mediated Wnt/β-catenin signaling, cell proliferation, and terminal differentiation

**Fig. 2:**
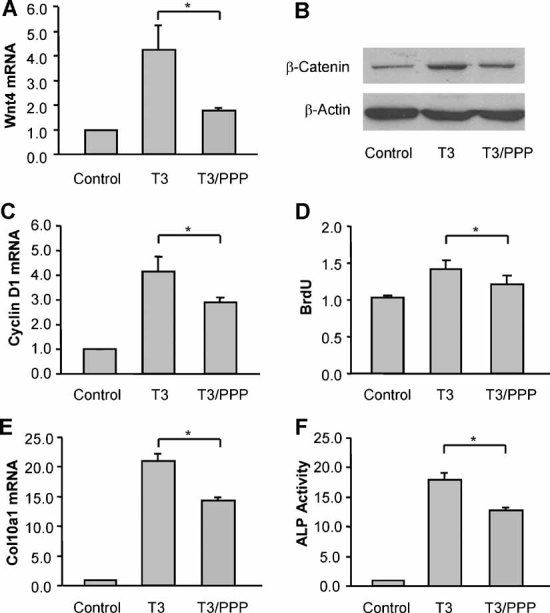
The inhibitor of IGF1R decreases T_3_-induced Wnt/β-catenin activation, cell proliferation, and terminal differentiation in growth plate chondrocytes. (*A*) Quantitative RT-PCR analysis of *Wnt4* mRNA expression in growth plate chondrocytes treated with T_3_ (100 ng/mL) and/or the IGF1R inhibitor picropodophyllin (PPP, 1 µM) for 5 days. The expression in T_3_-treated cells was normalized to the expression in control cells. (*B*) Immunoblotting of active β-catenin protein from whole-cell lysates of growth plate chondrocytes treated with T_3_ and/or PPP for 7 days. Actin was used as an internal control. (*C*) Detection of BrdU incorporation of growth plate chondrocytes treated with T_3_ and/or PPP for 5 days. (*D*) *Cyclin D1* mRNA expression in growth plate chondrocytes treated with T_3_ and/or PPP for 5 days. (*E*, *F*) *Col10a1* mRNA and alkaline phosphatase activity in growth plate chondrocytes treated with T_3_ and/or PPP for 5 days. *p < .05 versus the cells treated with T_3_ alone.

Picropodophyllin (PPP) is an inhibitor of the IGF1R tyrosine phosphorylation.(21) PPP inhibited T_3_-induced increases of *Wnt4* mRNA expression (Fig. 2*A*) and cellular accumulation of active β-catenin (Fig. 2*B*). PPP decreased T_3_-mediated cell proliferation, as assessed by BrdU incorporation (Fig. 2*C*) and *cyclin D1* mRNA expression (Fig. 2*D*). PPP also inhibited the T_3_-mediated hypertrophy of growth plate chondrocytes, as assessed by *Col10a1* mRNA expression (Fig. 2*E*) and ALP activity (Fig. 2*F*).

### IGF-1/IGF1R promotes Wnt/β-catenin signaling activation, cell proliferation, and terminal differentiation

**Fig. 3:**
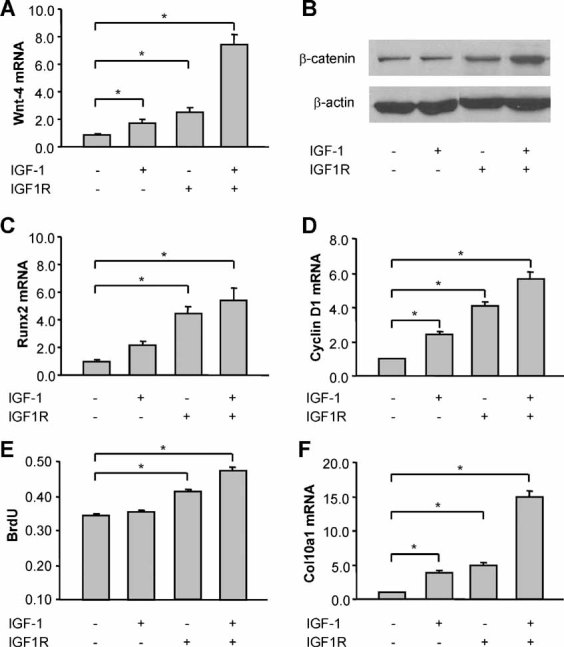
IGF-1/IGF1R promotes Wnt/β-catenin signaling, cell proliferation, and terminal differentiation of growth plate chondrocytes. (*A*) Quantitative RT-PCR analysis of *Wnt4* mRNA expression in rat growth plate chondrocytes infected with Ad-IGF1R or control adenovirus at an MOI of 50 and treated for 5 days with or without IGF-1 (50 ng/mL). (*B*) Immunoblotting of β-catenin from whole-cell lysates of growth plate chondrocytes treated with or without Ad-IGF1R and IGF-1 for 5 days. Actin was used as an internal control. (*C*, *D*) *Runx2* mRNA and *cyclin D1* mRNA expression in growth plate chondrocytes treated with Ad-IGF1R and/or IGF-1 for 5 days. (*E*) BrdU incorporation in growth plate chondrocytes treated with Ad-IGF1R and/or IGF-1 for 5 days. (*F*) *Col10a1* mRNA in growth plate chondrocytes treated with Ad-IGF1R and/or IGF-1 for 8 days. The data were expressed as the fold increase over the results of the cells infected with control adenovirus. *p < .05 versus control cells.

Incubation of the Ad-IGF1R-infected growth plate cells with 50 ng/mL of recombinant IGF-1 protein for 5 days upregulated the expression of *Wnt4* mRNA (Fig. 3*A*), increased cellular accumulation of β-catenin (Fig. 3*B*), and stimulated the expression of the Wnt/β-catenin response genes *Runx2*/*cbfa1* (Fig. 3*C*) and *cyclin D1* (Fig. 3*D*) in the growth plate chondrocytes. IGF-1 and Ad-IGF1R also increased BrdU incorporation by 5 days of treatment (Fig. 3*E*) and increased *Col10a1* mRNA expression by 8 days of treatment (Fig. 3*F*).

### Wnt antagonists attenuate the effects of IGF-1/IGF1R on β-catenin signaling, cell proliferation, and terminal differentiation

**Fig. 4:**
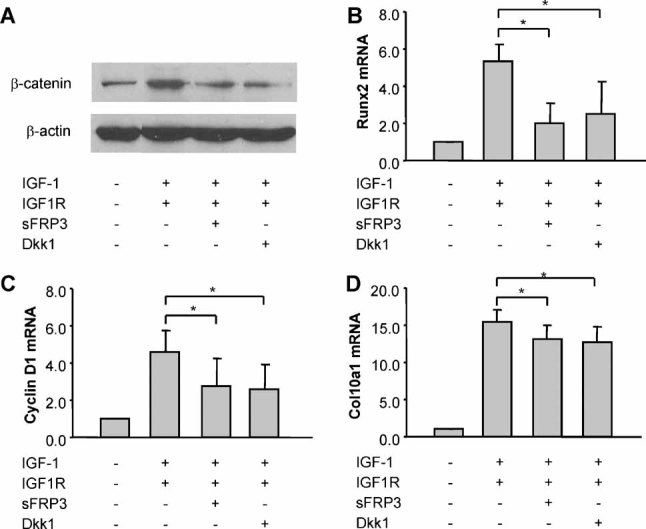
The effects of IGF-1/IGF1R on β-catenin signaling, cell proliferation, and terminal differentiation of growth plate chondrocytes are suppressed by inhibition of Wnt signaling. (*A*) Immunoblotting of β-catenin from whole-cell lysates of growth plate chondrocytes treated with Ad-IGF1R (MOI of 50) and IGF-1 (50 ng/mL) in the presence or absence of Wnt antagonists sFRP3 (100 ng/mL) and Dkk1 (100 ng/mL) for 5 days. (*B*, *C*) Quantitative real-time RT-PCR of *Runx2* mRNA (*B*) and *cyclin D1* mRNA expression (*C*) in rat growth plate chondrocytes treated with Ad-IGF1R and IGF-1 for 5 days in the presence or absence of sFRP3 and Dkk1. (*D*) *Col10a1* mRNA expression in growth plate chondrocytes treated with Ad-IGF1R and IGF-1 for 8 days in the presence or absence of sFRP3 and Dkk1. *p < .05 versus the cells treated with Ad-IGF1R and IGF-1.

The stimulatory effects of IGF-1/IGF1R on growth plate chondrocytes were partially reversed by the Wnt antagonists Frzb/sFRP3 and Dkk1 at concentrations of 100 ng/mL. Both sFPR3 and Dkk1 suppressed the IGF-1/IGF1R-mediated induction of active β-catenin (Fig. 4*A*), *Runx2* mRNA, *cyclin D1* mRNA, and *Col10a1* mRNA expression (Fig. 4*B–D*).

### T_3_ promotes the PI3K/Akt signaling and PI3K/Akt-induced β-catenin activation

**Fig. 5:**
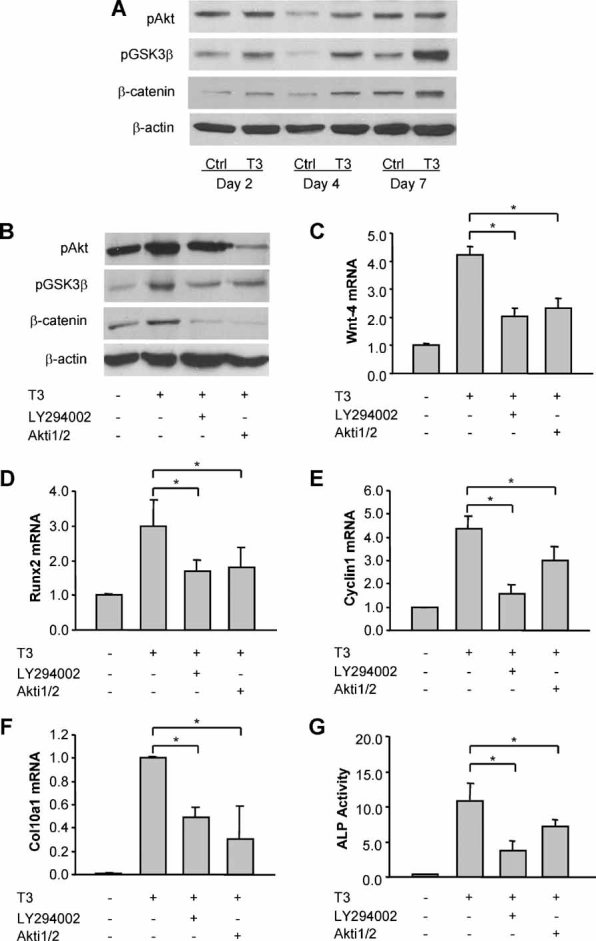
Thyroid hormone promotes IGF-1-activated PI3K/Akt signaling and PI3K/Akt-dependent β-catenin signaling in growth plate chondrocytes. (*A*) Immunoblotting of phosphorylated Akt (pAkt), phosphorylated GSK-3β (pGSK-3β), and active β-catenin protein from whole-cell lysates of growth plate chondrocytes treated with or without T_3_ (100 ng/mL) for 2 to 7 days. Actin was used as an internal control. (*B*) Immunoblotting of pAkt, pGSK-3β, and β-catenin protein of growth plate chondrocytes treated with T_3_ for 5 days in the presence or absence of PI3K signaling inhibitor LY294002 (20 µM) and Akt signaling inhibitor Akti1/2 (1 µM). (*C–E*) *Wnt4* mRNA (*C*), *Runx2* mRNA (*D*), and *cyclin D1* mRNA (*E*) expression of growth plate chondrocytes treated with T_3_ for 5 days in the presence or absence of LY294002 and Akti1/2. ^*^p < .05 versus the cells treated with T_3_ alone. (*F*, *G*) *Col10a1* mRNA (*F*) and ALP activity (*G*) of growth plate cells treated with T_3_ for 5 days in the presence or absence of LY294002 and Akti1/2. *p < .05 versus the cells treated with T_3_ alone.

To determine whether the effects of thyroid hormone also were mediated by the IGF-1-dependent PI3K/Akt pathway, we analyzed the activity of PI3K/Akt signaling by measuring the phosphorylation of Akt and its downstream target GSK-3β. Immunoblotting demonstrated increases in both pAkt and pGSK-3β on day 4 of T_3_ treatment (Fig. 5*A*), in parallel with the increase of *Igf1r* expression by T_3_ treatment (Fig. 1*B*). The level of pGSK-3β also was increased on day 7 in conjunction with the induction of active β-catenin (Fig. 5*A*).

In addition, we incubated T_3_-treated growth plate chondrocytes with the PI3K inhibitor LY294002 and the Akt inhibitor Akti1/2. The addition of 20 µM LY294002 or 1 µM Akti1/2 led to significant suppression of the T_3_-mediated increases in pGSK-3β and active β-catenin in growth plate chondrocytes (Fig. 5*B*). Both inhibitors also reversed the stimulatory effects of T_3_ on *Wnt4*, *Runx2*, and *cyclin D1* mRNA expression in the growth plate chondrocytes (Fig. 5*C–E*).

T_3_-induced increases in *Col10a1* mRNA expression and ALP activity were inhibited when growth plate cells were treated with T_3_ in the presence of LY294002 or Akti1/2 for 5 days (Fig. 5*F, G*). However, both LY294002 and Akti1/2 had no significant effect on the hypertrophic markers when added to the cells previously treated with T_3_ alone for 5 days (data not shown).

## Discussion

These experiments performed in pellet culture systems show that *Igf1r* expression is induced by thyroid hormone in proliferating and prehypertrophic cells, the same population of cells that express *Igf1r* in growth plate cartilage in vivo.(12) *Igf1* is expressed at much lower levels in growth plate chondrocytes than in perichondrium and metaphyseal bone. IGF-1 protein in the in vivo growth plate therefore is considered to derive primarily from surrounding perichondrium and bone than from the chondrocytes themselves.(18) Thyroid hormone therefore may regulate IGF-1 signaling in growth plate cells predominantly at the receptor level, although T_3_ treatment also leads to a transient increase in both *Igf1* mRNA expression and IGF-1 protein release. The inhibition of T_3_ action by the IGF1R inhibitor PPP supports a functional interaction between thyroid hormone and IGF-1 signaling in the regulation of growth plate chondrocyte growth and differentiation.

Our data demonstrate that IGF-1/IGF1R induces *Wnt4* expression and β-catenin activation and also stimulates growth plate chondrocyte proliferation and modestly promotes chondrocyte hypertrophy. However, the effects of IGF-1/IGF1R on terminal differentiation are limited and not comparable in magnitude with the effects of thyroid hormone treatment, suggesting that the effects on hypertrophy consequently are more likely to result from increasing the number of proliferating cells recruited from resting cells. IGF-1/IGF1R actions on growth plate chondrocytes are neutralized by the Wnt antagonists sFRP3 and Dkk1, confirming that the Wnt/β-catenin signaling pathway is downstream of IGF-1 signaling.

The PI3K/Akt pathway has been reported to mediate IGF-1-stimulated proliferation and differentiation in rat growth plate chondrocytes and chondrogenic cell lines.(22–24) The PI3K pathway is also required for normal growth plate differentiation and regulates endochondral bone growth.(25) The delayed bone ossification observed in *Akt1/Akt2* double-knockout mice is also similar to the phenotype of *Igf1r* knockout mice.(26)

Our data show that T_3_ treatment stimulates PI3K/Akt/GSK-3β signaling. The inhibition of PI3K/Akt activity by LY294002 and Akti1/2 prevents T_3_-induced *Wnt4* expression and β-catenin activation. PI3K/Akt signaling may potentiate the activation of Wnt pathways through the inactivation of GSK-3β and stimulation of Wnt ligand expression. LY294002 and Akti1/2 also prevent T_3_-induced growth plate chondrocyte differentiation but are unable to suppress terminal differentiation in cells committed to hypertrophy. PI3K/Akt signaling therefore may indirectly augment cell differentiation by promoting cell proliferation and recruiting more cells undergoing hypertrophy.

These results indicate that IGF-1 is actively involved in crosstalk between the Wnt/β-catenin and PI3K/Akt pathways in the regulation of growth plate chondrocyte proliferation and differentiation by thyroid hormone. Thyroid hormone receptors (TRα) are predominantly expressed in resting zone (RZ) and proliferating zone (PZ) growth plate chondrocytes,(27) whereas *Igf1r* is expressed in PZ and prehypertrophic zone (preHZ) growth plate cells,(12) and Wnt ligands are located primarily in preHZ and hypertrophic zone (HZ) cells.(28) We speculate that thyroid hormone binds to its nuclear receptor in RZ and PZ cells and initiates a series of events that lead the RZ cells to differentiate into proliferating and hypertrophic cells. Thyroid hormone initially may stimulate IGF-1/IGF1R signaling in PZ growth plate chondrocytes, activating the β-catenin signaling pathway through either the PI3K/Akt pathway or the Wnt pathway, in turn regulating the transcription of β-catenin-responsive genes (eg, *cyclin D1*) and subsequently promoting cell proliferation in the growth plate.

Thyroid hormone–activated Wnt/β-catenin signaling is more potent than IGF-1 signaling in promoting terminal differentiation of growth plate chondrocytes. IGF-1/IGF1R may indirectly augment chondrocyte hypertrophy by potentiating cell proliferation through the PI3K/Akt pathway, synergizing with the Wnt signaling pathway in regulating the maturation of growth plate chondrocytes.

**Fig. 6:**
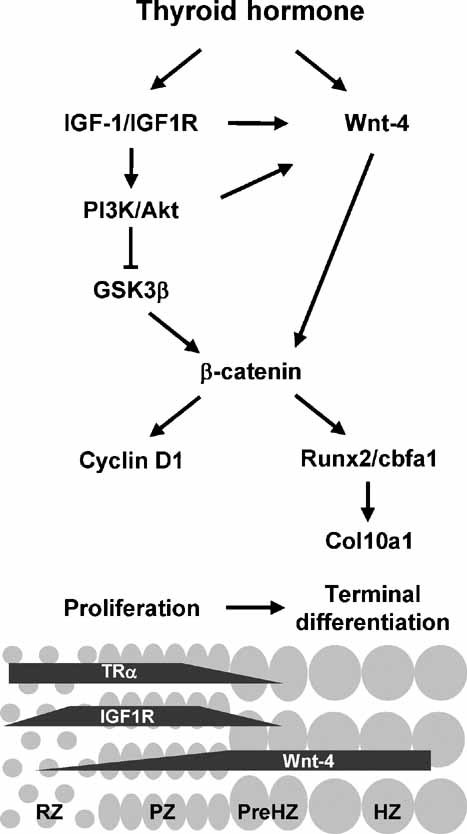
Schematic diagram of the proposed interactions between thyroid hormone, IGF-1/IGF1R, and β-catenin signaling pathways in regulating cell proliferation and terminal differentiation of growth plate chondrocytes. RZ = resting zone; PZ = proliferating zone; preHZ = prehypertrophic zone; HZ = hypertrophic zone.

The proposed interactions of these signaling pathways are illustrated in Fig. 6. In conclusion, thyroid hormone promotes growth plate cell differentiation and longitudinal bone growth by activating β-catenin signaling via modulation of IGF-1/IGF1R signaling through the Wnt and PI3K/Akt pathways. In addition to the IGF-1/IGF1R, PI3K/Akt, and Wnt signaling pathways, there are likely to be other pathways involved in the thyroid hormone–mediated effects on growth plate chondocytes because treatment with a combination of IGF1R inhibitors, PI3K/Akti inhibitors, and Wnt antagonists has additive but not complete inhibitory effects on the T_3_-induced terminal differentiation (data not shown).

## References

[1] Kronenberg HM (2003). Developmental regulation of the growth plate. Nature..

[2] Church V, Nohno T, Linker C, Marcelle C, Francis-West P (2002). Wnt regulation of chondrocyte differentiation. J Cell Sci..

[3] Hartmann C, Tabin CJ (2000). Dual roles of Wnt signaling during chondrogenesis in the chicken limb. Development..

[4] Wang L, Shao YY, Ballock RT (2007). Thyroid hormone interacts with Wnt/β-catenin signaling pathway in the terminal differentiation of growth plate chondrocytes. J Bone Miner Res..

[5] Hunziker EB, Wagner J, Zapf J (1994). Differential effects of insulin-like growth factor I and growth hormone on developmental stages of rat growth plate chondrocytes in vivo. J Clin Invest..

[6] Abbaspour A, Takata S, Matsui Y, Katoh S, Takahashi M, Yasui N (2008). Continuous infusion of insulin-like growth factor-I into the epiphysis of the tibia. Int Orthop..

[7] Mushtaq T, Bijman P, Ahmed SF, Farquharson C (2004). Insulin-like growth factor-I augments chondrocyte hypertrophy and reverses glucocorticoid-mediated growth retardation in fetal mice metatarsal cultures. Endocrinology..

[8] Wang Y, Nishida S, Sakata T (2006). Insulin-like growth factor-I is essential for embryonic bone development. Endocrinology..

[9] Baker J, Liu JP, Robertson EJ, Efstratiadis A (1993). Role of insulin-like growth factors in embryonic and postnatal growth. Cell..

[10] Liu JP, Baker J, Perkins AS, Robertson EJ, Efstratiadis A (1993). Mice carrying null mutations of the genes encoding insulin-like growth factor I (*Igf1*) and type 1 IGF receptor (*Igf1r*). Cell..

[11] Wang J, Zhou J, Bondy CA (1999). Igf1 promotes longitudinal bone growth by insulin-like actions augmenting chondrocyte hypertrophy. FASEB J..

[12] Parker EA, Hegde A, Buckley M, Barnes KM, Baron J, Nilsson O (2007). Spatial and temporal regulation of GH-IGF-related gene expression in growth plate cartilage. J Endocrinol..

[13] Firth SM, Baxter RC (2002). Cellular actions of the insulin-like growth factor binding proteins. Endocr Rev..

[14] O'Shea PJ, Bassett JH, Sriskantharajah S, Ying H, Cheng SY, Williams GR (2005). Contrasting skeletal phenotypes in mice with an identical mutation targeted to thyroid hormone receptor α1 or β. Mol Endocrinol..

[15] Chen M, Zhu M, Awad H (2008). Inhibition of β-catenin signaling causes defects in postnatal cartilage development. J Cell Sci..

[16] Gordon MD, Nusse R (2006). Wnt signaling: multiple pathways, multiple receptors, and multiple transcription factors. J Biol Chem..

[17] Brazil DP, Yang ZZ, Hemmings BA (2004). Advances in protein kinase B signalling: AKTion on multiple fronts. Trends Biochem Sci..

[18] Raucci A, Bellosta P, Grassi R, Basilico C, Mansukhani A (2008). Osteoblast proliferation or differentiation is regulated by relative strengths of opposing signaling pathways. J Cell Physiol..

[19] Playford MP, Bicknell D, Bodmer WF, Macaulay VM (2000). Insulin-like growth factor 1 regulates the location, stability, and transcriptional activity of β-catenin. Proc Natl Acad Sci USA..

[20] Ballock RT, Reddi AH (1994). Thyroxine is the serum factor that regulates morphogenesis of columnar cartilage from isolated chondrocytes in chemically defined medium. J Cell Biol..

[21] Vasilcanu D, Girnita A, Girnita L, Vasilcanu R, Axelson M, Larsson O (2004). The cyclolignan PPP induces activation loop-specific inhibition of tyrosine phosphorylation of the insulin-like growth factor-1 receptor: link to the phosphatidyl inositol-3kinase/Akt apoptotic pathway. Oncogene..

[22] Kiepe D, Ciarmatori S, Hoeflich A, Wolf E, Tönshoff B (2005). Insulin-like growth factor (IGF)–I stimulates cell proliferation and induces IGF binding protein (IGFBP)-3 and IGFBP-5 gene expression in cultured growth plate chondrocytes via distinct signaling pathways. Endocrinology..

[23] Ciarmatori S, Kiepe D, Haarmann A, Huegel U, Tönshoff B (2007). Signaling mechanisms leading to regulation of proliferation and differentiation of the mesenchymal chondrogenic cell line RCJ3.1C5.18 in response to IGF-I. J Mol Endocrinol..

[24] Phornphutkul C, Wu KY, Yang X, Chen Q, Gruppuso PA (2004). Insulin-like growth factor-I signaling is modified during chondrocyte differentiation. J Endocrinol..

[25] Ulici V, Hoenselaar KD, Gillespie JR, Beier F (2008). The PI3K pathway regulates endochondral bone growth through control of hypertrophic chondrocyte differentiation. BMC Dev Biol..

[26] Peng XD, Xu PZ, Chen ML (2003). Dwarfism, impaired skin development, skeletal muscle atrophy, delayed bone development, and impeded adipogenesis in mice lacking *Akt1* and *Akt2*. Genes Dev..

[27] Robson H, Siebler T, Stevens DA, Shalet SM, Williams GR (2000). Thyroid hormone acts directly on growth plate chondrocytes to promote hypertrophic differentiation and inhibit clonal expansion and cell proliferation. Endocrinology..

[28] Andrade AC, Nilsson O, Barnes KM, Baron J (2007). Wnt gene expression in the post-natal growth plate: regulation with chondrocyte differentiation. Bone..

